# Timing of orchidopexy and its relationship to postoperative testicular atrophy: results from the ORCHESTRA study

**DOI:** 10.1093/bjsopen/zraa052

**Published:** 2021-02-13

**Authors:** Paediatric Surgical Trainees Research Network (PSTRN), Organizing and Writing Group:, C Skerritt, C Bradshaw, N Hall, L McCarthy, M Woodward

## Abstract

**Background:**

In 2011 a consensus statement from the British Association of Paediatric Urologists recommended lowering the age at orchidopexy to under 1 year. There are concerns that a younger age at operation may increase postoperative testicular atrophy. The ORCHESTRA study aimed to establish the current age at orchidopexy in a multicentre, international audit and to see whether testicular atrophy was affected by age at operation.

**Methods:**

The study was undertaken over a 3-month period in 28 centres in boys undergoing orchidopexy for unilateral, palpable undescended testes. Data collection was done using a standardized, predetermined protocol. The primary outcome was postoperative testicular atrophy. Secondary outcomes were wound infections, reoperations, and unplanned hospital stays related to anaesthetic events.

**Results:**

A total of 417 patients were included, of whom only 48 (11.5 per cent) underwent orchidopexy before 1 year of age. There was no difference in anaesthetic complications in boys aged less than 1 year *versus* older patients: 0 of 48 (0 per cent) *versus* 6 of 369 (1.6 per cent) (*P* = 0.999). Complete follow-up was available for 331 patients (79.4 per cent). There was no difference in atrophy rate between those aged less than 1 year and older boys: 1 of 37 (3 per cent) *versus* 9 of 294 (3.1 per cent) (*P* = 0.999). Reoperation rates were 0 of 37 (0 per cent) and 7 of 294 (2.4 per cent) respectively (*P* = 1.000). There were more wound infections in boys under 1 year of age: 4 of 37 (11 per cent) *versus* 7 of 294 (2.4 per cent) (*P* = 0.025).

**Conclusion:**

Only 11.5 per cent of boys underwent surgery before the age of 1 year. There was no increased risk of postoperative testicular atrophy with early surgery, although there was a higher rate of wound infection. Further study is required to demonstrate that early orchidopexy is not inferior to orchidopexy undertaken in boys aged over 1 year.

## Teaser

This was a prospective, international study of the effect of age at orchidopexy on postoperative testicular atrophy. Only 11.5 per cent of boys underwent surgery at age below 1 year as recommended by British Association of Paediatric Urologists guidelines. The rate of testicular atrophy did not vary according to age, but there was a significantly increased risk of wound infection in boys operated at under 1 year of age.

## Resumen

**Antecedentes:**

En 2011, una declaración de consenso de la *British Association of Paediatric Urologists* recomendó reducir la edad de la orquidopexia por debajo de 1 año. Existe la preocupación de que una edad más temprana en el momento de la operación pueda aumentar la atrofia testicular postoperatoria. El estudio ORCHESTRA tuvo como objetivo establecer la edad actual de la orquidopexia en una auditoría internacional multicéntrica y evaluar si la atrofia testicular se ve afectada por la edad en el momento de la operación.

**Métodos:**

El estudio se realizó durante un período de 3 meses en 28 centros en niños sometidos a orquidopexia por testículos no descendidos palpables unilaterales. La recogida de datos se realizó utilizando un protocolo predeterminado y estandarizado. El objetivo primario fue la atrofia testicular postoperatoria. Los objetivos secundarios fueron las infecciones de la herida, las reintervenciones y las estancias hospitalarias no planificadas relacionadas con eventos anestésicos.

**Resultados:**

Se incluyeron 417 pacientes de los cuales solo 48 (11,5%) fueron sometidos a orquidopexia antes del año de edad. No hubo diferencia en las complicaciones anestésicas en los niños menores de un año *versus* los pacientes mayores (0/48 (0%) *versus* 6/294 (1,6%)), *P* = 0,999. Se dispuso de seguimiento completo en 331 (80%) pacientes. No hubo diferencia en la tasa de atrofia en los menores de un año (1/37 (2,7%)) frente a los niños mayores (9/294) (3,1%)), *P* = 0,999. Las tasas de reintervención fueron 0/37 en < 1 año *versus* 7/294 (2,7%) en > 1 año, *P* = 0,999. Hubo más infecciones de heridas en niños menores de un año (4/37 (10,5%)) en comparación con los pacientes mayores (7/294 (2,4%)), *P* = 0,025.

**Conclusión:**

Solo el 11,5% de los niños se sometieron a cirugía antes de cumplir 1 año. No hubo un mayor riesgo de atrofia testicular postoperatoria para la cirugía temprana, aunque hubo una mayor tasa de infección de la herida. Se requieren más estudios para demostrar que la orquidopexia temprana no es inferior a la orquidopexia realizada a partir de un año de edad.

## Introduction

In September 2011 the British Association of Paediatric Urologists (BAPU) published a consensus statement recommending that orchidopexy should be performed in boys from as young as 3 months of age, although surgery at 6–12 months was considered acceptable^1^. This was based on evidence that operating at an earlier age may improve fertility and reduce the long-term risk of testicular malignancy^2–5^. It also assumed that undescended testes that have not descended spontaneously by 3 months of age are unlikely to do so by 1 year^6^. Since then, other professional bodies^7^^,^^8^ have issued similar guidance supporting early orchidopexy, and a recent meta-analysis^9^ concluded that the ideal age for orchidopexy was 6–12 months.

There is, however, a lack of evidence to ensure that changes in practice do not have unforeseen negative consequences. Surgery for undescended testes may result in testicular atrophy, and it is not known whether operating at a younger age may increase the risk of postoperative testicular atrophy^10^. There is also heightened awareness of the possible detrimental effects of general anaesthesia on the developing brain, resulting in a US Food and Drug Administration safety communication about use of general anaesthetics in children aged less than 3 years^11^.

The ORCHESTRA (ORCHidopexy: does Earlier Surgery affect Testicular Atrophy?) study was designed as a multicentre, international audit of current practice of orchidopexy in relation to BAPU guidance, and established current rates of testicular atrophy after orchidopexy.

## Methods

The study was a multicentre, international trainee-led prospective audit of practice led by the UK-based Paediatric Surgical Trainees Research Network (PSTRN). Data were recorded from an inception cohort of boys undergoing orchidopexy for unilateral, palpable undescended testis. Data were collected from each centre over 3 months and outcomes recorded for boys with at least 6 months of follow-up. Results are reported in accordance with the STROBE guidelines^12^. No preregistration exists for the study reported in this article.

### Centre eligibility

Any hospital that provided elective general paediatric surgery in the UK was eligible to enter patients. International centres were contacted through trainee links to these countries. In the UK, trainees ensured that there was consultant oversight and agreement in departments to participate. Each centre registered the study locally as a clinical audit. International centres followed local ethical approval procedures. Data collection was completed by surgical trainees.

### Patient eligibility

All boys aged less than 16 years in whom orchidopexy was performed for unilateral, palpable undescended testis were eligible for inclusion. The testis was considered palpable either during preoperative clinical examination or during examination under general anaesthesia. Patients who required laparoscopy to determine testicular position were excluded. Boys who had a known endocrine or genetic condition that could affect testicular growth, and those who underwent orchidopexy following ipsilateral inguinal hernia repair were also excluded.

### Study outcomes

The audit standard was defined as the proportion of boys who received orchidopexy before 12 months of age, and was set at 100 per cent. The age at referral was also registered to determine whether delays in timing of orchidopexy were attributable to late referral.

The primary postoperative outcome was the rate of testicular atrophy at least 6 months after orchidopexy. The audit standard was set at less than 5 per cent testicular atrophy based on a recently published large retrospective case series^10^. Testicular atrophy was measured in comparison with the contralateral, normally descended testis. At the time of orchidopexy, the surgeon was asked to note the volume of the testis as a proportion of the contralateral testis using one of the following categories: less than 25 per cent; 25 per cent or more but less than 50 per cent; 50 per cent or more but less than 75 per cent; 75 per cent or more but less than 100 per cent; 100 per cent; or more than 100 per cent. At follow-up, the clinician was again asked to note the testicular size compared with the contralateral testis. Testicular atrophy was defined as a greater than 50 per cent decrease in size to allow for the fact that this was a pragmatic and subjective measurement, and that small differences may be due to inter-rater variability and differences in time interval between assessments.

Secondary outcomes included: rate of reoperation/testicular ascent, defined by a testis that was palpable at follow-up but no longer in a scrotal position and deemed to require further surgery, where the audit standard was set at less than 2 per cent^13^; a surgical-site infection rate less than 2 per cent where infection was defined by purulent drainage from the incision or at least two of: pain or tenderness, localized swelling, redness, heat, fever diagnosed by the clinician as representing a surgical-site infection, or organisms and pus cells from an aspirate or wound swab (data were collected on whether patients were given antibiotic treatment for presumed wound infection)^14^; and an unplanned overnight stay as a result of an adverse anaesthetic event, where the standard was also set at less than 2 per cent^15^.

### Data collection

Data were collected for each eligible patient using a standardized online electronic pro forma. Patients were identified on a weekly basis from planned elective lists. Preoperative data such as age at referral and operative data were registered by or with the operating surgeon at the end of the list. Follow-up information was collected at the outpatient clinic at least 6 months after orchidopexy.

Anonymized patient data were entered into an online database via a secure webpage. Each data collector was granted password access, and a study number was allocated once an individual patient data collection form had been completed. Trainees entering the data then kept a secure record on the hard drive of a hospital network computer linking the study number to a hospital number used for follow-up. The website generated automatic e-mail reminders when follow-up was planned.

### Study size design and bias

A local audit was performed at the lead centre (Oxford Children’s Hospital), which performs about 100 orchidopexies per year, to inform the study design. Of these, about 20 per cent are for intra-abdominal or bilateral undescended testes. A 3-month data collection period was chosen as a balance between achieving reasonable numbers from each centre (estimated at 20 per paediatric surgical centre) and a short enough time frame for enthusiasm for the study to be sustained. The plan was to recruit 10 paediatric surgical centres and recruit 200 patients over 3 months. In addition, recognizing that more than half of all orchidopexies in the UK are performed in district general hospitals^16^, often by adult general surgeons or urologists with a specialist interest in general paediatric surgery, the study aimed to recruit 20 district hospitals through the national research collaborative of adult surgical trainees to obtain a further 200 patients, based on data from a moderately sized hospital that reported about 35 orchidopexies per year^17^.

### Statistical analysis

Planned statistical analysis included comparing postoperative outcomes between patients operated at less than 1 year of age compared with older patients, using Fisher’s exact test or χ^2^ test for categorical data. Only patients who had completed at least 6 months of follow-up were included in the outcome analysis.

## Results

Some 417 patients were included from 28 centres (13 paediatric surgical centres in the UK, 11 general hospitals in the UK, 4 international paediatric centres in Argentina, Austria, Finland, and Lithuania). A further five district general hospitals that registered to take part in the audit did not perform any orchidopexies during the data collection period.

Results were available on age at referral for 356 boys (85.4 per cent). The median age at referral was 1 year 8 months (range 1 day to 15 years 1 month). Of the 356 boys, 117 (32.9 per cent) were referred before 1 year of age. Of the 417 boys, 92 (22.1 per cent) had previous documentation of intrascrotal testes by a health professional before diagnosis of an undescended testis and referral.

**Fig. 1:**
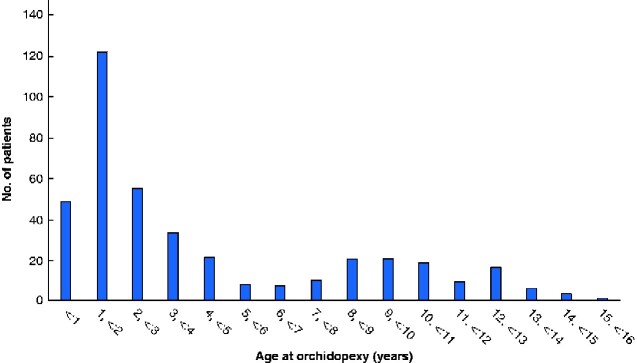
Age at orchidopexy

The median age at surgery was 2 years 6 months (range 3 months to 15 years 4 months), although there was a bimodal distribution with a peak between 1 and 2 years of age, and a further smaller peak at around 10 years (*Fig. 1*). The median age at surgery for boys who had ascending testes was 6 years 1 month; when these boys were excluded, the median age at surgery for those with congenital undescended testis was 2 years.

Only 48 of 417 boys (11.5 per cent) underwent orchidopexy before 1 year of age. Of the 28 centres, 17 treated boys who were aged less than 12 months at the time of orchidopexy. All of the overseas centres performed orchidopexies in boys aged less than 12 months, as did 11 of the 13 UK paediatric surgical centres, but only 2 of the 11 general hospitals. When those with ascending testes were excluded, the proportion of boys who received surgery before 1 year of age was 14.4 per cent.

### Surgical details

Some 356 patients (85.4 per cent) had surgery in the UK, 58 (16.3 per cent) in general hospitals and 298 (83.7 per cent) in paediatric surgical centres.

In UK specialist centres, the primary operator was a consultant in 30.2 per cent of operations whereas in non-specialist centres the primary operator was a consultant 84 per cent of the time. In specialist centres in the UK and overseas, unsupervised trainees performed 11.4 and 16 per cent of procedures respectively (*Table 1*).

**Table 1:** Operating grade of primary surgeon

	UK paediatric surgical centre (*n* = 298)	UK district general hospital (*n* = 58)	International paediatric surgical centre (*n* = 61)
Consultant	90 (30.2)	49 (84)	30 (49)
Trainee, supervised	174 (58.4)	9 (16)	21 (34)
Trainee, unsupervised	34 (11.4)	0 (0)	10 (16)

Values in parentheses are percentages.

Twenty-five boys (6.0 per cent) had an undescended testis that was impalpable until they were anaesthetized; 60.0 per cent of undescended testes (250 of 417) were right-sided. Some 376 boys (90.2 per cent) underwent surgery via a groin incision and a separate scrotal incision, whereas 41 (9.8 per cent) had a single scrotal incision.

As the study was limited to orchidopexies for testes that were palpable under general anaesthesia, 160 (38.4 per cent) were found within the inguinal canal, 231 (55.4 per cent) in the superficial inguinal pouch below the external ring of the inguinal canal, and 26 (6.3 per cent) had an ectopic location (3 perineal, 8 lateral to the scrotum, 5 lateral to the external ring, and 10 described as ectopic but without a precise location) (*Table 2*).

**Table 2:** Location of testis before orchidopexy

	No. of testes
	(*n* = 417)
Inguinal	160 (38.4)
Superficial inguinal pouch	231 (55.4)
Ectopic	26 (6.3)

Values in parentheses are percentages.

At the time of orchidopexy, 323 out of 417 (77.5 per cent) of undescended testes were recorded to be at least 75 per cent of the volume of the contralateral testis. Only 9 per cent were less than half the volume of the contralateral testis. In 86 patients (20.6 per cent) the undescended testis was noted to have a dissociated vas.

#### Testicular atrophy

Follow-up at 6 months after orchidopexy was available for 331 patients (79.4 per cent). Of these, 10 (3.0 per cent) had an atrophic testis (reduction in size compared with time of operation of more than 50 per cent). There was no significant difference in testicular atrophy between those operated before 12 months and those who had surgery after 12 months of age: 1 of 37 (3 per cent) *versus* 9 of 294 (3.1 per cent) (*P* = 1.000) (*Table 3*). Among operations performed by a trainee without consultant supervision, only one patient (2 per cent) developed testicular atrophy.

**Table 3:** Early postoperative complications after orchidopexy according to age at operation

	Age at operation		
	≤ 12 months	**> 12 months**	P*
	(*n* = 37)	(*n* = 294)	
**Testicular atrophy**	1 (3)	9 (3.1)	1.000
**Testicular re-ascent (redo orchidopexy)**	0 (0)	7 (2.4)	1.000
**Surgical-site infections**	4 (11)	7 (2.4)	0.025

Values in parentheses are percentages. *Fisher’s exact test.

#### Testicular re-ascent

Seven testes (2.1 per cent) were noted to have ascended after surgery. None of the patients with testicular re-ascent underwent orchidopexy before 12 months of age. A further 12 patients (3.6 per cent) were booked for further follow-up because their testes were high in the scrotum and there was concern that they may need further surgery. None of the testicular re-ascents were in patients operated via a single trans-scrotal incision.

#### Anaesthetic complications

There were six unplanned overnight admissions (1.4 per cent). In two patients, apnoeic events occurred in recovery, one patient was slow to wake, two required additional pain relief (1 had undergone circumcision under the same general anaesthetic), and one patient did not pass urine for several hours and also had issues with pain management. None of infants under 12 months of age had an unplanned overnight admission.

#### Surgical-site infections

There were more wound infections in younger boys: 4 of 37 (11 per cent) operated under 12 months of age *versus* 7 of 294 older boys (2.4 per cent); this was statistically significant (p = 0.025).

## Discussion

This study did not show an increase in testicular atrophy among boys who had surgery at less than 12 months of age, suggesting that current guidance is not associated with worse early postoperative outcomes. The 3.0 per cent rate of testicular atrophy is less than the preset audit standard of 5 per cent, and similar to that reported in recent observational studies^10^^,^^18^^,^^19^, which ranged from 2.6 to 5.0 per cent.

The rate of testicular re-ascent was similar in boys operated at less than 12 months and those operated after 12 months. There was a 2.1 per cent rate of reoperation during follow-up, in line with the audit standard of 2 per cent. Although there were no cases of testicular re-ascent in boys operated before 12 months of age, this result was not statistically significant reflecting the small numbers in this group. There may be advantages to operating at a younger age because the testis is moved a shorter distance in absolute terms. However, a further 12 patients (3.6 per cent) remained under review owing to concerns that the testis was lying high in the scrotum and redo orchidopexy might be needed. A weakness of the study was that longer follow-up was not obtained to check that the redo surgery rate was not higher than that reported.

In orchidopexies performed via a single trans-scrotal incision, reported rates of testicular re-ascent vary from 0 to 4.5 per cent^19^^,^^20^. There were no testicular re-ascents in boys who underwent orchidopexy via a single trans-scrotal incision in the present study, supporting its use in selected patients.

Anaesthetic complication rates were also similar in both groups, and there was a low overall rate of unplanned postoperative admissions of 1.4 per cent, compared with the audit standard of less than 2 per cent. There were no adverse anaesthetic events recorded in boys operated under 1 year of age. However, the APRICOT study^21^, which included over 30 000 children who had a general anaesthetic, found that there was a 12 per cent decreased risk of anaesthetic complication for each additional year of age. A weakness of the present study was that no data were collected on intraoperative anaesthetic events.

In the present study, there was a significantly increased risk of surgical-site infection requiring antibiotics in boys under 12 months of age. In this age group, the wounds are more likely to be exposed to urine and faecal contamination from wearing nappies. It may be that the younger boys would benefit from an occlusive dressing or wound glue application^22^.

There are several limitations to this study. It was hoped to include more non-specialist centres in the UK through the adult surgical trainee research networks, but only 16 centres were recruited. A much smaller number of orchidopexies was undertaken in each district hospital than predicted and several registered hospitals performed no orchidopexies during the study interval. The majority of orchidopexies were done in specialist centres and, although they showed equivalent outcomes in different age groups, it is not clear whether this would be the case outside specialist centres. Only four boys under the age of 1 year had surgery in non-specialist centres. In the UK this has implications for the design of treatment pathways for undescended testes. Until recently, most orchidopexies were performed in non-specialist centres; however, there has been a trend towards more children receiving surgery in specialist centres and trying to achieve a younger age at orchidopexy may further accelerate this^16^^,^^23^^,^^24^.

Despite most orchidopexies being performed in specialist centres, only 11.5 per cent of procedures in this study were undertaken before 12 months of age, and this meant the comparisons of outcomes involved very unequal numbers of patients. The reasons for older age at orchidopexy appeared to be multifactorial, including delayed referral, waiting times, and ascending testes. In this cohort, 22.1 per cent of boys had an undescended testis that had been documented previously as intrascrotal by a health professional. This is not dissimilar to the rate in a multicentre study^25^ in Germany, which found that 27 per cent of orchidopexies were performed for ascending testis. In the UK, baby checks are carried out routinely at 6–8 weeks of age by primary care physicians. The prevalence of cryptorchidism has been reported to fluctuate during the first year of life, starting at 5.9 per cent of newborns and dipping to 2.7 per cent at 3 months, before unexpectedly rising again to 6.7 per cent at 1 year of age^26^. Baby checks may well take place when more testes are palpable in the scrotum, but a proportion of these ascend over time. The latest edition of *Health for All Children*^27^, which describes best practice for health screening in primary care, emphasizes urgent referral for bilateral undescended testes found at the 6-week check, but makes no recommendation for management of unilateral undescended testes. The recent commissioning guide for paediatric orchidopexy^28^ published by the Royal College of Surgeons of England recommends referral at 6 months of age for undescended testes.

Only 79.4 per cent of the patients in this study had follow-up documented at 6 months following surgery. As the study was registered as a prospective audit of practice, direct contact with study participants outside of the usual standard of care was not permitted. A study^29^ that looked at factors associated with non-attendance at follow-up reported a similar 80 per cent attendance rate for orchidopexy follow-up visits in the Canadian healthcare system. The loss to follow-up of some 20 per cent of patients means that there was systematic bias in the outcome reporting of postoperative testicular atrophy. As the baseline demographics of patients with or without follow-up were similar (*Table S1*), it seems likely that that parents would be more inclined to attend follow-up if they had ongoing concerns, so underestimation of the rate of atrophy seems unlikely.

Testicular volume was assessed clinically by the surgical team responsible for the patient, leading to observer bias and inter-rater variability. The study protocol minimized these risks by requesting that testicular volume was compared with that of the contralateral testis, and that the volume had to decrease by two groups (over 50 per cent difference) in the classification system to count as testicular atrophy. There is a lack of consensus regarding what constitutes testicular atrophy. Ein and colleagues defined testicular atrophy as a loss of volume of more than one-third compared with the contralateral testis^10^, whereas Carson *et al.*^18^deemed that more than 50 per cent loss of volume counted as testicular atrophy. Both sets of data were collected retrospectively from chart reviews relying on physician descriptions of the testis and it is unclear how testicular volume was assessed or consistently documented. The ORCHESTRA study had clear definitions of how to assess and classify testicular size. This, along with contemporary data collection, should have improved the robustness of the data.

The pragmatic design of the study is reflective of standard clinical practice and so the results should be generalizable. There was no restriction on the operating grade of the surgeon or the surgical approach. The results did not show worse outcomes in patients who had been operated on by trainees, and support unsupervised trainee operating when trainers consider their trainees competent to perform an orchidopexy.

Robust evidence is still needed to influence clinicians to refer boys at an earlier age for consideration of orchidopexy, and to satisfy surgeons that there is no increased risk of early postoperative complications. The present study did not show an increased risk of postoperative testicular atrophy in boys who had surgery at less than 12 months of age, suggesting that current guidelines are safe, despite the current low rate of compliance.

## Collaborators

Organizing and Writing Group: C. Skerritt (Bristol Royal Hospital for Children, Bristol, UK); C. J. Bradshaw (Bristol Royal Hospital for Children, Bristol, UK); N. J. Hall (Southampton Children’s Hospital, Southampton, UK); L. S. L. McCarthy (Birmingham Children’s Hospital, Birmingham, UK); M. N. Woodward (Bristol Royal Hospital for Children, Bristol, UK). Collaborators: J. Ng, C. Healy (Royal London Hospital, London, UK); H. Ghattaura, J. Wells, P. Sarmah (Birmingham Children’s Hospital, Birmingham, UK); B. S. Allin (Oxford Children’s Hospital, Oxford, UK); N. J. Wright, C. Richardson (Evelina Children’s Hospital, London, UK); Z. Haveliwala (Manchester Children’s Hospital, Manchester, UK); S. Jaunoo (University Hospital Coventry and Warwickshire, Coventry, UK); N. F. Walker (Kingston University Hospital, Kingston upon Thames, UK); M. Horridge (Sheffield Children’s Hospital, Sheffield, UK); A. R. Ross (Norfolk and Norwich Hospital, Norwich, UK); E. Fishleigh (Sandwell and West Birmingham Hospital, Birmingham, UK); J. Frae, S. Tiboni (Leicester Children’s Hospital, Leicester, UK); A. Salim, R. Jeeneea (Alder Hey Hospital, Liverpool, UK); K. Cao (North Middlesex Hospital, London, UK); S. Prodan (Hospital Italiano de Buenos Aires, Buenos Aires, Argentina); R. Roberts, R. Clark (Bristol Royal Hospital for Children, Bristol, UK); A. Getman (Kaunas Clinic, Kaunas, Lithuania); J. R. Davidson (University Hospital Lewisham, London, UK); D. Mullasery (Addenbrooke’s Hospital, Cambridge, UK); B. Lakshminarayanan, J. Durell (Southampton University Hospital, Southampton, UK); P. Jackson (Nottingham Hospital, Nottingham, UK); J. Inglis (Royal Wolverhampton Hospital, Wolverhampton, UK); F. Friedmacher (University Hospital Graz, Graz, Austria); P. King, M. Hotston, K. Kumaresan (Royal Cornwall Hospital, Truro, UK); A. Joshi (Broomfield Hospital, Chelmsford, UK); E. Smyth (Musgrove Park Hospital, Taunton, UK); S. Shiralkar (Russells Hall Hospital, Dudley, UK); N. Pakkasjarvi (Helsinki Hospital, Helsinki, Finland); S. Arman (Basildon Hospital, Basildon, UK).

## Supplementary Material

zraa052_Supplementary_Data

## Notes

Members of the Paediatric Surgical Trainees Research Network (PSTRN) are co-authors of this study and are listed under the heading Collaborators

Presented to a meeting of the British Association of Paediatric Surgeons, July 2017, London
