# Supplementary figures and images for: Timing of orchidopexy and its relationship to postoperative testicular atrophy: results from the ORCHESTRA study
Source: BJS Open. 2021 Feb 13;5(1):zraa052. doi: 10.1093/bjsopen/zraa052 (PMC7893476; doi:10.1093/bjsopen/zraa052)

**Table S1 Characteristics of patients with and without follow-up
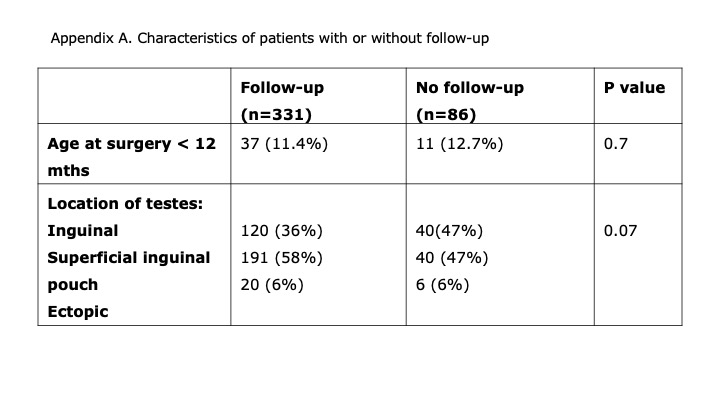
**

Supplement: zraa052_Supplementary_Data [file zraa052_supplementary_data.docx]
